# Does gradual change in head positioning affect cerebrovascular physiology?

**DOI:** 10.14814/phy2.13603

**Published:** 2018-02-08

**Authors:** Man Y. Lam, Victoria J. Haunton, Thompson G. Robinson, Ronney B. Panerai

**Affiliations:** ^1^ Department of Cardiovascular Sciences University of Leicester Leicester United Kingdom; ^2^ National Institute for Health Research Leicester Biomedical Research Centre University of Leicester Leicester United Kingdom

**Keywords:** Cerebral autoregulation, head positioning, reproducibility

## Abstract

We studied cerebral blood velocity (CBV), and associated hemodynamic parameters during gradual changes in head positioning in a nonstroke group. CBV (transcranial Doppler ultrasound), beat‐to‐beat blood pressure (BP, Finometer), and end‐tidal carbon dioxide (ETCO_2_, capnography) were recorded between lying flat (0°) and sitting up (30°) head positions, in 18 volunteers (10 female, mean age, 57 ± 16 years), at two visits (12 ± 8 days). A significant reduction was found between 5‐min FLAT (0°) and 5‐min SIT (30°) positions in CBV (visit 1: 4.5 ± 3.3%, *P* = 0.006; visit 2: 4.1 ± 3.5%, *P* = 0.003), critical closing pressure (CrCP; visit 1: 15.5 ± 14.0%, *P* = 0.0002; visit 2: 14.1 ± 7.8%, *P* = 0.009) and BP (visit 1: 8.3 ± 7.4%, *P* = 0.001; visit 2: 11.0 ± 11.3%, *P* < 0.001). For 5 min segments of data, the autoregulation index and other hemodynamic parameters did not show differences either due to head position or visit. For 30 sec time intervals, significant differences were observed in the following: (BP,* P* < 0.001; dominant hemisphere (DH) CBV,* P* < 0.005; nondominant hemisphere (NDH) CBV,* P* < 0.005; DH CrCP,* P* < 0.001; NDH CrCP,* P* < 0.001; DH resistance area product (RAP), *P* = 0.002; NDH RAP,* P* = 0.033). Significant static changes in BP, CBV and CrCP, and large transient changes in key hemodynamic parameters occur during 0° to 30°, and vice versa, with reproducible results. Further studies are needed following acute ischemic stroke to determine if a similar responses is present.

## Introduction

Cerebral autoregulation (CA) is an important mechanism whereby cerebral perfusion is normally maintained at a constant level, over a relatively wide BP range (Aaslid et al. 1989; Paulson et al. 1990). Dynamic cerebral autoregulation (dCA) is usually assessed by looking at the response of cerebral blood flow (CBF) following a rapid, and transient alteration in perfusion pressure, usually over a period of seconds (Aaslid et al. 1989). Using a standard hospital bed, changing head position from lying flat (0°) to sitting up (30°) could be a straightforward technique in assessing dCA, and would be particularly useful for participants who are medically unstable, difficult to mobilize and/or with cognitive impairment or communication difficulty to ensure compliance with more complex protocols, for example: acute ischemic stroke (AIS) patients.

Traditionally, AIS patients are nursed with elevation of the head (≥30°), aiming to prevent aspiration pneumonia, and to reduce intracranial pressure (ICP), thereby avoiding hypertension‐related reperfusion damage (Kenning et al. 1981). The *A Very Early Rehabilitation Trial (AVERT)* (Bernhardt et al. 2015) was a multicenter, randomized controlled trial reporting that intensive early mobilization (upright position) was associated with unfavorable 3‐month functional outcome. A possible mechanism is that early mobilization, particularly in severe stroke (i.e., NIHSS>16), may result in reduced CBF secondary to acute stroke‐associated impairment in CA, adversely impacting on penumbral viability and consequently clinical outcome (Olavarria et al. 2014).

However, there is surprisingly little literature, with inconsistent findings on the impact of changes in head positioning on cerebral hemodynamics and associated parameters. Moreover, there is limited information about whether such changes persist following changes in head positioning. Some studies were inconclusive (Ropper et al. 1982; Fan 2004), while others favored lying flat (Rosner and Coley 1986; Wojner‐Alexander et al. 2005; Olavarria et al. 2014), or head elevation (Kenning et al. 1981; Winkelman 2000). In AIS patients, Wojner‐Alexander et al. (2005) reported an approximate 20% increase in middle cerebral artery (MCA) mean velocity when head position changed from 30° to 0°. However, this study did not separately report on the affected and unaffected hemispheres. Hunter et al. (2011) only observed impaired CBF in the affected hemisphere (in the incompletely recanalized group), but not the completely recanalized group or unaffected hemisphere, whereas Aries et al. (2013) suggested there was significant CBV reduction in the affected hemisphere, when head position changed from lying flat to upright (70°), but not the half‐sitting position (45°). Though approximately 50% of AIS patients received intravenous thrombolysis in this latter study, no comment on any difference between the completed and incompletely recanalized groups was made.

To understand cerebral hemodynamic changes in response to head positioning in an AIS population, it is first important to study a nonstroke control group to determine the normal physiological response. Therefore, the aim of this study was to investigate the extent of CBV change, and associated cerebral hemodynamic parameters, specifically autoregulation index (ARI), resistance area product (RAP) and critical closing pressure (CrCP), during a gradual change from a lying flat (0°) to sitting up (30°) head position, in a nonstroke control group, and over two visits in order to assess reproducibility.

In this study, we tested the hypothesis that changes in CA, and associated hemodynamic parameters, are developed during gradual changes in head positioning and such changes persist. We also tested the hypothesis that the above findings could be reproduced between visits. To test the hypothesis, we measured the CBV by transcranial Doppler ultrasound (TCD), before, during and after gradual change in head positioning in a nonstroke control group.

## Methods

### Participants

The study was carried out in accordance with the Declaration of Helsinki (2013) of the World Medical Association and was approved by the Wales Research Ethics Committee 1 (Reference: 15/WA/0328).

Volunteers were recruited from the University of Leicester and by poster advertisement at the University Hospitals of Leicester NHS Trust (UHL). All participants had no history of cerebrovascular disease and had given written informed consent. Additional exclusion criteria included participants who practiced yoga regularly, and female participants who were pregnant, lactating or planning pregnancy during the course of the study. Female participants who were on premenopause and currently on any form of contraception, during menstruation or postmenopause were eligible. Participants who had mild, controlled hypertension or any other cardiovascular disease were accepted as representative of active, but otherwise healthy, older participants.

### Procedure

The study was carried out in a dedicated research laboratory, which was at a controlled temperature (20–24^°^C) and free from distraction. Prior to attending the assessment, each participant was asked to avoid excessive food intake, caffeine, alcohol, and strenuous exercise for at least four hours prior to each recording. Effort was made to ensure all assessments were carried out in the morning and at a similar time of day at both visits. Beat‐to‐beat noninvasive blood pressure (BP) was recorded continuously using the Finometer^®^ device (Finapres Medical Systems; Amsterdam, Netherlands) attached to the middle finger of the nondominant hand. The finger with the cuff on was held at heart level to minimize any orthostatic pressure differences between finger and heart. Simultaneous bilateral insonation of the MCAs was performed using TCD (Viasys Companion III, Natus Medical Incorporated, California, USA), with a 2‐MHz probe. A head frame was used to secure the ultrasound probes in position and to minimize their movement. The proximal segment of the MCA was identified according to the depth, waveform, and characteristics of the signal (Aaslid et al. 1982). The depth, power, velocity, and location of the temporal windows were documented in each participant to ensure the same measurement parameters were used at the subsequent visit. Heart rate was recorded using a 3‐lead electrocardiogram (ECG). Respiratory rate was recorded, and ETCO_2_ was measured using small nasal cannula (Salter Labs, California, USA) attached to an infra‐red capnograph (Capnocheck Plus, Smith Medical, Minnesota, USA).

**Figure 1:**
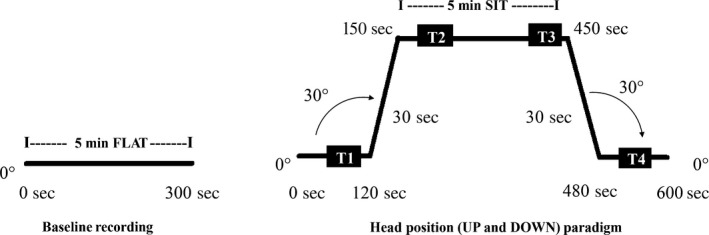
Diagram representing 5‐min FLAT and SIT periods, and selected time points (*T*1, *T*2, *T*3, and *T*4) used to extract mean parameter values in the head position paradigm with gradual elevation of the head from 0° to 30° and return to horizontal.

All parameters were recorded over a 5‐min baseline (5‐min FLAT) recording period, with the participant in the lying flat (0°) head position on a standard hospital bed. The recumbent angle was measured with an electronic goniometer built in‐house (Department of Medical Physics, UHL, Leicester, United Kingdom). Thereafter, another 2‐min recording was carried out while the participant was still in the lying flat (0°) head position; afterward, the recumbent angle was changed from 0° to 30° over 30 sec (UP phase); another 5‐min recording was carried out while participants were in the 30° head position (5‐min SIT); thereafter, the recumbent angle was changed from 30° back to 0° over 30 sec (DOWN phase); another 2‐min recording was carried out when the participant was in the lying flat (0°) head position (Fig. 1). This maneuver was repeated twice at each visit.

### Data analysis

Data were simultaneously recorded onto a physiological data acquisition system (PHYSIDAS, Department of Medical Physics, UHL NHS Trust, Leicester, United Kingdom) for subsequent off‐line analysis. All signals were digitized at 500 samples·sec^−1^ and data editing was subsequently carried out using in‐house software. The BP signal was calibrated at the beginning of each recording. Calibration was performed using the brachial BP readings taken by an OMRON 705IT sphygmomanometer. BP waveforms were then inspected for any drift and all signals were visually inspected to identify any artifacts and noise; narrow spikes (<100 msec) were removed by linear interpolation. Data were excluded from analysis if there was excessive artifact, noise or poor signal quality.

A median filter was applied to the CBV signal, and all signals were filtered using a zero‐phase, low pass Butterworth filter, with a cuff‐off frequency of 20 Hz. The R‐R interval was automatically marked from the ECG tracing, and continuous HR was plotted against time. Any ectopic beats, resulting in spikes in the HR signal, were identified by visual inspection. These were then manually corrected by remarking R‐R intervals for the time points at which they occurred. In cases where there was ECG drift or noise, cardiac cycles were marked using the BP tracing as an alternative. Mean, systolic and diastolic BP and CBV, were calculated for each cardiac cycle, as well as HR. Critical closing pressure (CrCP) and resistance area product (RAP) were estimated using the first harmonic method (Panerai 2003); these parameters being calculated to reflect the instantaneous CBV‐BP relationship changes that accompany changes in head positioning. Linear interpolation was used to obtain estimates of ETCO_2_ synchronized to the end of each cardiac cycle. Mean BP and CrCP were compensated for changes in head height by subtracting DH‐T*0.735*sin30° where DH‐T is the heart to temporal window distance in each subject. Beat‐to‐beat data were spline interpolated, with resampling at 5 Hz to produce signals with a uniform time base.

Mean values of all cerebral and peripheral hemodynamic variables, lasting 30 sec were extracted, these were expressed as time point 1 (*T*1) (80–110 sec), time point 2 (*T*2) (160–190 sec), time point 3 (T3) (400–430 sec) and time point 4 (*T*4) (490–520 sec), respectively. The 5‐min FLAT and SIT periods and time points *T*1–*T*4 are represented in Figure 1.

The autoregulation index (ARI), ranging from 0 (absence of autoregulation) to 9 (best observed CA), was estimated as described previously (Tiecks et al. 1995; Brodie et al. 2009). In summary, the CBV response to a hypothetical step change in BP was derived by transfer function analysis (TFA), using the parameter setting recommended by the International Cerebral Autoregulation Research Network (Claassen et al. 2016). Each of the 10 template CBV step responses proposed by Tiecks et al. (1995) was compared with the estimated step response and the ARI value corresponding to the best fit, assessed by the minimum mean square error, was adopted for each of the 5 min recordings corresponding to the FLAT (0°) or SIT (30°) positions. The main advantage of using ARI as compared to using either gain or phase from TFA is that it automatically incorporates the influences of both gain and phase, for the entire frequency response, without the need to select specific frequency bands.

### Statistical analysis

Data were assessed for normality using the Shapiro‐Wilk test. All normally distributed data are presented as mean ± standard deviation, and continuous skewed data are presented as median [interquartile range].

A paired *t*‐test or Wilcoxon sign‐ranked test was adopted to identify any inter‐hemispheric differences. In the absence of inter‐hemispheric differences, values were averaged from both hemispheres. For those participants who only had unilateral measurements, that value was used. As baseline 0° (5‐min FLAT) recordings were performed once and the 10‐min gradual head positioning paradigm (HPP) was performed twice in each visit, the gradual HPP readings were averaged prior to statistical comparison with the baseline 0° (5‐min FLAT) reading.

Two‐way repeated measures ANOVA was undertaken to assess the effects of head position (5‐ min FLAT vs. 5‐min SIT), time (*T*1–*T*4) and reproducibility (visit 1 vs. visit 2) separately. The Tukey post hoc test was used to perform individual comparisons when ANOVA showed significant effects. A significance level of *P* ≤ 0.05 was adopted for all results.

## Results

Eighteen participants (10 female) were recruited with a mean age of 57 ± 16 years (range 26–87), 3 participants were aged <50 years old. Suitable transtemporal windows could not be found in two participants who were then removed from the study. Four participants only had a single transtemporal window (two right, two left). According to the Edinburgh Handedness Inventory (Oldfield 1971), 15 participants were right‐handed and one participant was left‐ handed. Details of other participant demographic parameters are reported in Table 1.

**Table 1:** Demographic characteristics of participants

Participants (*n*)	16
Age (years)	57 ± 16
Sex (female: male)	8:8
Handedness (right: left)	15:1
Body mass index (BMI) kg.m^−2^	24 ± 4
Smoker (*n*)	1
Yes	2
Ex	13
No	
Past medical history	
Hypertension	5
Medication	
Antihypertensive therapy	5

### Baseline data

Baseline CBV values at visit 1 were 53.9 ± 15.6 cm·sec^−1^ and 53.0 ± 12.6 cm·sec^−1^ for the dominant (DH) and nondominant hemispheres (NDH), respectively. Corresponding values for visit 2 were 52.4 ± 12.3 cm·sec^−1^ and 51.3 ± 12.7 cm·sec^−1^. There were no significant differences in mean baseline CBV values between visit 1 and visit 2 for either hemisphere (DH, *P* = 0.57; NDH, *P* = 0.41). In addition, no significant differences in inter‐hemispheric CBV baseline values were found for the 12 subjects who had bilateral recordings (1st visit, *P* = 0.42; 2nd visit, *P* = 0.38). Therefore, averaged values were used in subsequent analyses. No significant differences were found for peripheral parameters (BP, HR, and ETCO_2_) at baseline between the two visits (Table 2).

**Table 2:** Peripheral and cerebral hemodynamic parameters for FLAT (0°) and SIT (30°) head positions for two repeated visits

Parameter	Visit 1		Visit 2		*P* value (head position effect)	*P* value (visit effect)
	5‐min FLAT (0°)	5‐min SIT (30°)	5‐min FLAT (0°)	5‐min SIT (30°)		
BP (mmHg)	89.1 ± 12.1	81.8 ± 8	89.6 ± 11.3	79.8 ± 11.4	<0.001	0.39
Heart rate (bpm)	65.7 ± 9.9	65.1 ± 9.7	65.3 ± 11.3	65.1 ± 11	0.42	0.89
End‐tidal CO_2_ (mmHg)	38.4 ± 4.7	37.3 ± 4.6	39.3 ± 3	39.5 ± 1.7	0.39	0.38
CBV (cm·sec^−1^)	53.5 ± 13.6	51.1 ± 13.8^a^	51.9 ± 12.2	49.7 ± 12.9^b^	<0.0005	0.66
CrCP (mmHg)	39 ± 10.4	32.5 ± 9.2^a^	40.1 ± 11	32.9 ± 11.4^b^	<0.0005	0.77
RAP (mmHg·sec·cm^−1^)	0.94 ± 0.31	1.02 ± 0.32	0.95 ± 0.27	0.99 ± 0.32	0.13	0.93
ARI	5.64 ± 1.42	5.47 ± 1.1	6 ± 1.37	6.12 ± 1	0.86	0.1

Values are mean ± SD for 5 min segments of data. *P* values from two‐way ANOVA for the difference between 5‐min FLAT and SIT head positions and between visits 1 and 2. BP, blood pressure; bpm, beats per minute; CBV, cerebral blood velocity; CrCP, critical closing pressure; RAP, resistance area product; ARI, autoregulation index.

a Tukey post hoc compared to FLAT head position *P* < 0.05 in visit 1.

b Tukey post hoc compared to FLAT head position *P* < 0.05 in visit 2.

### Effects of head position (5‐min FLAT and SIT)

Peripheral and cerebral hemodynamic parameters averaged over 5‐min FLAT and SIT head positions are given in Table 2 for each visit. BP, CBV, and CrCP values were reduced in the SIT head position at both visits 1 and 2, respectively, (BP: 8.3 ± 7.4% and 11.0 ± 11.3%, *P* < 0.001; CBV: 4.5 ± 3.3% and 4.1 ± 3.5%, *P* < 0.0005; CrCP: 14.1 ± 7.8% and 15.5 ± 14.0%, *P* < 0.0005). No significant differences in ARI (*P* = 0.86; *P* = 0.1) and other peripheral or cerebral hemodynamic parameters, averaged over 5 min, were seen due to head position or between visits, respectively, (Table 2).

### Temporal pattern of peripheral and cerebral hemodynamic responses

**Figure 2:**
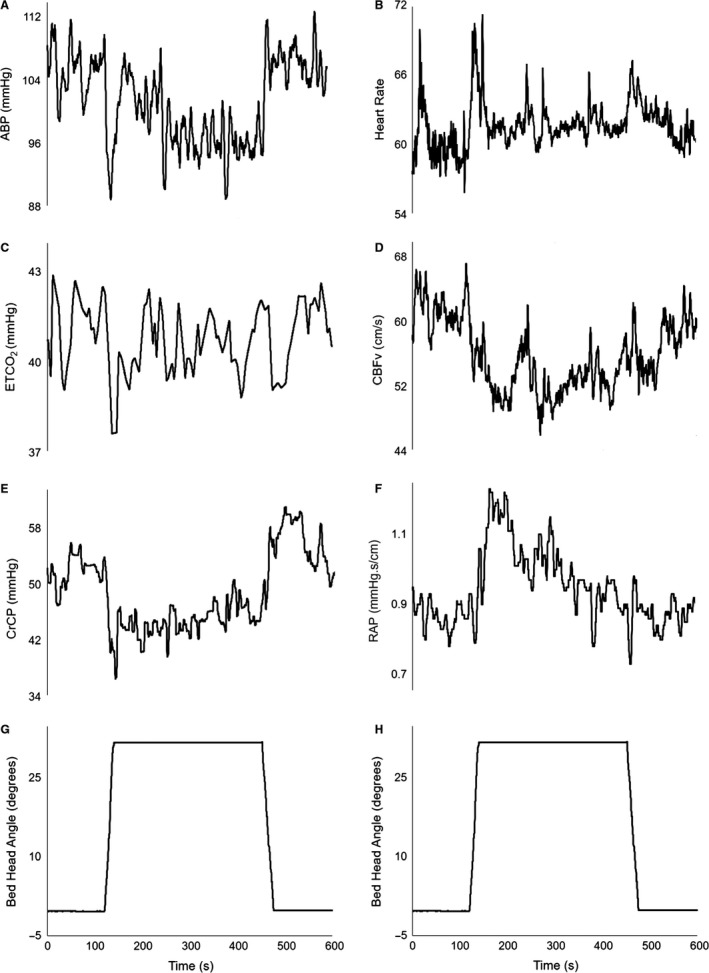
Representative recordings of BP (A), HR (B), ETCO_2_ (C), dominant hemisphere CBV (D), CrCP (E), RAP (F) during gradual change in head positioning, as indicated by the bed head angle (G).

**Figure 3:**
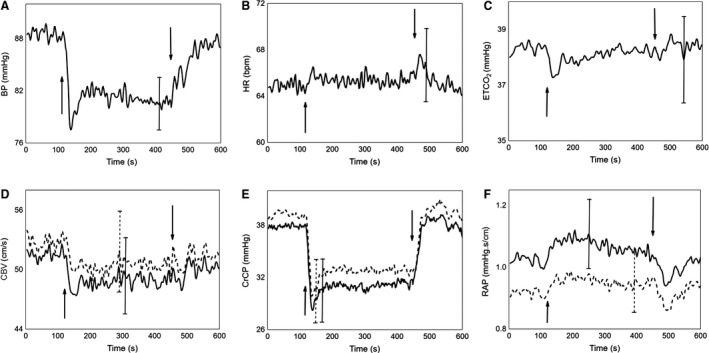
Population averages of BP (A), HR (B) and ETCO_2_ (C), CBV (D), CrCP (E) and RAP (F) during gradual changes of head positioning. Upward arrow shows the point when the head position changed from 0° to 30° (UP phase) and downward arrow from 30° to 0° (DOWN phase). Dominant hemisphere (continuous line) versus nondominant hemisphere (dotted line). For clarity, the error bar represents only the largest ± 1 SE at the point of occurrence.

**Figure 4:**
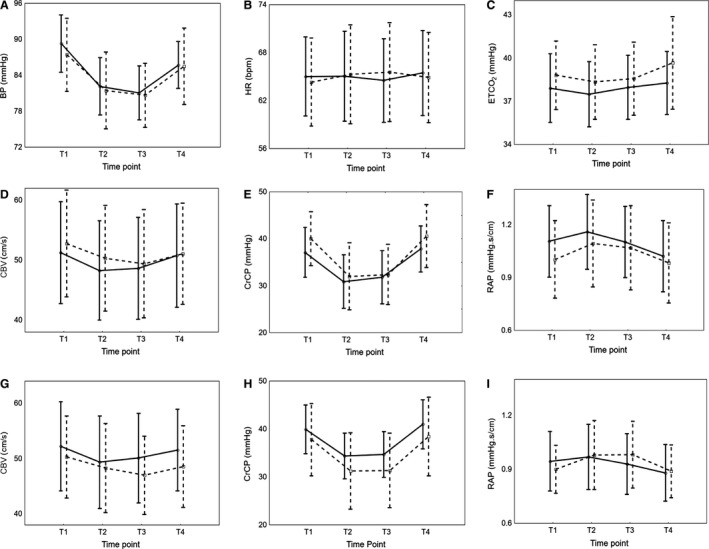
Effects of visit on the changes in BP (A), HR (B), ETCO_2_ (C), CBV (D and G), CrCP (E and H) and RAP (F and I). First visit (continuous line) versus second visit (dotted line). Dominant hemisphere (D, E, and F) versus nondominant hemisphere (G, H, and I). Vertical bar denotes 95% confidence interval.

Representative recordings during gradual change in head positioning are shown in Figure 2, and population averages are given in Figure 3. With the exception of HR and ETCO_2_, all other parameters showed significant changes across selected time points (*T*1–*T*4) for both visits (Table 3). Of note, most parameters showed relatively large transient changes during UP and DOWN phase, even in cases when the *T*1–*T*4 ANOVA did not indicate an overall significant *F*‐value (Fig. 4).

**Table 3:** Peripheral and cerebral hemodynamic parameters at selected time points (*T*1‐*T*4)

Parameter	Visit	*T*1	*T*2	*T*3	*T*4	*P* value (time point effect)	*P* value (visit effect)
BP (mmHg)	1	89.3 ± 8.6	82.2 ± 8.6^a^	81 ± 8.1^a^	85.7 ± 7.1^a^	<0.001	0.7
	2	87.4 ± 11	81.5 ± 11.6^a^	80.7 ± 9.7^a^	85.5 ± 11.5		
Heart rate (bpm)	1	65 ± 9.3	65.1 ± 10.6	64.5 ± 9.8	65.5 ± 10	0.76	1
	2	64.3 ± 10.4	65.3 ± 11.6	65.6 ± 11.6	64.9 ± 10.6		
End‐tidal CO_2_ (mmHg)	1	37.9 ± 4	37.5 ± 3.7	38 ± 3.7	38.3 ± 3.6	0.39	0.38
	2	38.8 ± 4	38.3 ± 4.3	38.6 ± 4.2	39.7 ± 5.3		
DH CBV (cm·sec^−1^)	1	51.2 ± 13.4	48.3 ± 13^a^	48.6 ± 13.4	50.7 ± 13.6	<0.005	0.77
	2	52.4 ± 12.9	49.3 ± 13^a^	48.7 ± 13.2	50.4 ± 12.3		
NDH CBV (cm·sec^−1^)	1	53.8 ± 14.7	51 ± 15.1^a^	51.9 ± 15.3	53.2 ± 13.4	<0.005	0.11
	2	50.7 ± 12.5	48.8 ± 13.5	47.6 ± 12^a^	48.9 ± 12.4		
DH CrCP (mmHg)	1	37.1 ± 8.8	30.9 ± 9.4^a^	31.8 ± 9.4^a^	37.9 ± 8.1	<0.001	0.68
	2	40 ± 9.5	32 ± 11.8^a^	32.4 ± 10.6^a^	40.6 ± 11.1		
NDH CrCP (mmHg)	1	39.9 ± 8.8	34.4 ± 8.3^a^	34.7 ± 8.2^a^	41 ± 8.9	<0.001	0.6
	2	37.8 ± 13	31.3 ± 13.9^a^	31.4 ± 13.5^a^	38.5 ± 14.2		
DH RAP (mmHg·sec·cm^−1^)	1	1.11 ± 0.34	1.16 ± 0.35	1.1 ± 0.33	1.02 ± 0.33^b^	0.002	0.56
	2	1 ± 0.36	1.09 ± 0.41	1.07 ± 0.4	0.98 ± 0.38^b^		
NDH RAP (mmHg·sec·cm^−1^)	1	0.94 ± 0.29	0.97 ± 031	0.93 ± 0.29	0.88 ± 0.27^b^	0.033	0.89
	2	0.9 ± 0.23	0.98 ± 0.33	0.98 ± 032	0.89 ± 0.25^b^		

Data are mean ± SD. *P*‐values from two‐way ANOVA for effects of time (*T*1–*T*4) and visit. BP, blood pressure; bpm, beats per minute; CBV, cerebral blood velocity; CrCP, critical closing pressure; RAP, resistance area product; ARI, autoregulation index; DH, dominant hemisphere; NDH, nondominant hemisphere.

a Tukey post hoc *P* < 0.05 compared to *T*1.

b Tukey post hoc *P* < 0.05 compared to *T*2.

During the UP phase (120 to 150 sec), CBV showed an initial bilateral steep decline (Fig. 3, DH 51.12 ± 13.61 to 47.37 ± 11.89 cm·sec^−1^; NDH 52.82 ± 14.49 to 49.75 ± 13.43 cm·sec^−1^; DH Tukey's *P* = 0.0005, NDH Tukey's *P* = 0.001) over 30 sec, followed by a plateau during the 5‐min SIT period (150 to 450 sec). During the DOWN phase (450 to 480 sec), there was a nonsignificant trend toward recovery, compared to the UP position (Fig. 3, DH 48.75 ± 12.64 to 50.11 ± 13.67 cm·sec^−1^; NDH 49.31 ± 12.91 to 52.36 ± 14.61 cm·sec^−1^).

### Reproducibility of the head position paradigm across two separate visits

Paradigm‐synchronized population averages are shown in Figure 4 for the selected *T*1–*T*4 time points at both visits 12 ± 8 days apart. Though ETCO_2_, CBV (NDH), CrCP (NDH), and RAP (DH), showed differences in baseline values, there were no significant differences in overall temporal patterns in any parameters between visits (Table 3).

## Discussion

### Main findings

We studied the effects of gradual change in head positioning, on both peripheral and cerebral hemodynamic parameters in a nonstroke control group, over two visits. BP, CBV, and CrCP were significantly reduced in the 5‐min SIT compared to 5‐min FLAT position. No other significant changes in peripheral or cerebral hemodynamics were detected, and this was consistent over both visits. However, over a 10‐min gradual HPP, significant transient changes were seen in the majority of peripheral and cerebral hemodynamic parameters, including CBV, RAP, and CrCP, particularly in association with the 30 sec UP and DOWN phases. Again, these changes were reproducible over two visits.

Though reproducibility of cerebral and circulatory responses to postural change in healthy subjects (Houtman et al. 1999), healthy older participants (Gabbett and Gass 2005), and patients with syncope (Sagristà‐Sauleda et al. 2002) have previously been reported, to the best of our knowledge, this is the first study to provide a detailed description of the cerebral and peripheral hemodynamic changes taking place during gradual HPP, including the reproducibility of key parameters before, during and after a gradual change in head positioning. Of considerable relevance, dCA was not affected by a change from 0° to 30° head position in this nonstroke control group, which is important for future longitudinal studies of such changes in a diseased population, including studies of AIS patients during the acute and recovery periods.

### Effects of head position on peripheral hemodynamics and ETCO_2_

In the absence of other similar studies in the literature, comparisons are only possible with related studies, such as changes in posture induced by tilt. These show broad general agreement with the temporal pattern of HR, BP, and ETCO_2_ during the 10‐min gradual HPP (Borst et al. 1982; Sprangers et al. 1991; Smith et al. 1994; Cencetti et al. 1997; Serrador et al. 2006). Though the present study recruited a wide age range group, the majority of participants were aged over 50, where the normal physiological responses to head position change are blunted in aging, with alterations in circulatory response, changes in autonomic function and reduction in the efficiency of the muscle pump; all contributing to a delay in compensatory mechanisms (Lakatta 1986; Narayanan et al. 2001). Interestingly, despite a BP drop during the UP phase, we did not observe a significant compensatory heart rate increase, which may relate to previous studies having a faster postural change (3 to 6 sec) (Smith et al. 1970; Sprangers et al. 1991) compared to our study (30 sec), and therefore may have reduced the magnitude of any physiological changes (Ewing et al. 1980). Furthermore, head‐up tilt (70°) and standing have previously been utilized (Borst et al. 1982; Sprangers et al. 1991; Cencetti et al. 1997), in contrast to the 0° to 30° changes used in our study, which are more typical of the changes seen in an acute medically unwell bed‐bound patient population.

A mild, but consistent, ETCO_2_ reduction is noted during both UP and DOWN phases. Hypocapnia secondary to posture change has been previously demonstrated in tilt studies (Cencetti et al. 1997; Serrador et al. 2006); with hyperventilation (Novak et al. 1998), reduction in venous return with reduced CO_2_ delivery to the lung (Gisolf et al. 2004), and total body blood flow redistribution (Anthonisen and Milic‐Emili 1966) hypothesized as possible explanations.

### Effects of head position on cerebral hemodynamics

CA is important in maintaining CBF during posture changes, through myogenic, metabolic, chemical, and neurogenic mechanisms (Heistad and Kontos 1983; Johnson 1989; Paulson et al. 1990). Rosner and Coley (1986) previously reported that even moderate head elevation could compromise CPP and therefore, CBF. The temporal pattern of CBV changes found in the present study was in keeping with former studies (Romero et al. 2011; Garrett et al. 2017). Romero et al. (2011) carried out a HUT study on nine healthy volunteers, during both euhydration and dehydration and found that the percentage of CBV reduction during HUT was consistent and statistically nonsignificant between both conditions. Garrett et al. (2017) carried out a study of 18 young healthy volunteers and observed a 13% reduction in CBV when changing from supine (0°) to seated (90°) posture.

CrCP represents the BP at which CBF ceases, when transmural pressure is not sufficient to counteract the active tension imposed by the smooth muscle, and vessels collapse with cessation in blood flow (Panerai 2003). It reflects ICP, arterial tone and the metabolic mechanisms involved in underlying CBF regulation. It is generally accepted that head elevation is associated with a reduction in ICP, in both healthy and disease states (Rosner and Coley 1986; Feldman et al. 1992; Meixensberger et al. 1997; Schwarz et al. 2002). RAP is an index of cerebrovascular resistance; the inverse of the linear CBV‐BP relationship slope, and has been suggested as an indicator of myogenic control (Panerai 2003; Panerai et al. 2005, 2012). The temporal pattern of RAP changes in response to head elevation (Figs. 2F and 3F) seems to support this interpretation where the gradual reduction in RAP, following a profound fall in BP, maintains a relatively constant CBV during the 5‐min head UP. Despite these phasic changes, no significant differences in RAP were observed between averaged 5‐min FLAT and SIT positions. This contrasts with a previous report by Robertson et al. (2014) who found a nonsignificant rise in RAP but significant CrCP reduction, particularly in healthy older adults during upright posture, suggesting that CrCP played a dominant role in response to posture change. Castro et al. (2012) recruited 13 healthy young volunteers, aged 26 ± 9 years, and performed a reading task in supine, sitting and head‐up tilt positions, and reported a significant reduction in MCA CBV, and an associated significant increase in CrCP from supine to head‐up tilt, but no such change in RAP.

ARI values were not influenced by postural change. Similarly, Lefthériotis et al. (1998) reported no ARI differences between supine and 40° head upright tilt positions, nor did (Carey et al. 2003) during 30‐min head‐up tilt in a young and older healthy volunteer population. To explain such diverse changes, with reductions in CBV, in the absence of CA changes, further work is needed to consider an integrated multivariate model, possibly including the contributions of PaCO_2_, ICP, and sympathetic nervous system activity.

### Clinical implications and future work

Previous studies have used a variety of techniques to investigate hemodynamic parameters in response to postural change, for example: supine to sitting (Fagard et al. 1994), squatting (Philips et al. 2012), standing (Ewing et al. 1978), or head‐up tilt (Youde et al. 2003). Uniquely, we used an electronic goniometer to record the degree of head position change on a standard hospital bed, which could enable medically unstable and cognitively impaired patients to comply with the protocol; ideal for AIS patient studies. In addition, the gradual HPP was sufficient to elicit measureable changes in peripheral and cerebral hemodynamic responses, which were reproducible. This will enable longitudinal changes to be investigated in disease populations, as well as the potential beneficial and detrimental effects of physiological manipulations in the acute and recovery periods of illness.

### Study limitations

Our study has a number of limitations. First, only nonstroke volunteers were included, though we did allow subjects with controlled chronic medical conditions, similar to those seen in an AIS population, to participate. Noteworthy, the ARI values for this group (Table 2), are in excellent agreement with values reported in the literature for healthy subjects (Tiecks et al. 1995; Patel et al. 2016) thus suggesting that our volunteers had normal dynamic CA. We included a wide age range (26–87 years) of healthy subjects, though only three participants were aged under 50 years old, which may confound the results of the study. However, van Beek et al. (2008), using a variety of methods to assess dCA in elderly populations, have not found an influence of aging on dCA. Second, a formal sample size calculation was not possible since no previous data were available from similar studies. However, our sample size of 16 participants would have an 80% power to detect an ARI difference of 2 units at the 5% significance level (Brodie et al. 2009). Accordingly, it is difficult to draw meaningful conclusions in respect of more subtle cerebral hemodynamic regulatory responses to gradual changes in head positioning. Therefore, with only 16 subjects, it is possible that we might have missed changes in ARI of less than 2 units. Therefore, future studies should focus on an older population with larger number of participants. Third, we did not explore within‐visit reproducibility, though we were able to demonstrate consistency of peripheral and cerebral hemodynamic responses between visits, including at four time‐points during the gradual HPP protocol. We worked under the assumption that intra‐visit reproducibility was very high and used repeated measurements to improve results, and to ensure at least one good set of data in each session. Finally, TCD only measures CBV and assumes that the insonated vessel diameter is constant, so reflecting CBF changes (Willie et al. 2011). Coverdale et al. (2014) have demonstrated the cross sectional area of MCA changes under hyper‐ and hypocapnic conditions, and therefore CBV may underestimate CBF in such situations. On the other hand, noninvasive arterial volume clamping for continuous BP recording has been found to correlate well with invasive BP assessments, providing consistent results in the head‐up tilt setting (Petersen et al. 1995; Sammons et al. 2007).

## Conclusions

Dynamic cerebral autoregulation, assessed over a 5 min time interval, was not affected by gradual changes in head position, but there were static changes in BP, CBV, and CrCP that were reproducible on repeated visits approximately 2 weeks apart. Of relevance, with the exception of HR and ETCO_2_, most other parameters (BP, CBV, CrCP, RAP) showed highly significant transient (dynamic) changes in response to head elevation or return to the 0° flat position. Understanding these cerebral and peripheral hemodynamic changes due to head position are critical for the management of critically ill patients, including AIS, severe head injury, following neurosurgery and in particular, conditions which are prone to develop elevated ICP. Furthermore work is needed to assess the impact of disease, including impairment of CA, on the physiological response to changes in head position.

## Conflict of Interest

No conflicts of interest, financial or otherwise, are declared by the author(s). TGR is an NIHR Senior Investigator.
